# Comparison of hyperthermia and adrenaline to enhance the intratumoral accumulation of cisplatin in a murin model of peritoneal carcinomatosis

**DOI:** 10.1186/1756-9966-30-4

**Published:** 2011-01-07

**Authors:** Olivier Facy, François Radais, Sylvain Ladoire, Delphine Delroeux, Hervé Tixier, François Ghiringhelli, Patrick Rat, Bruno Chauffert, Pablo Ortega-Deballon

**Affiliations:** 1 INSERM 866, Equipe Avenir, Dijon, France; 2 Department of Digestive Surgical Oncology, University Hospital of Dijon, France; 3 Department of Digestive Surgery, University Hospital of Besançon, France; 4 Department of Medical Oncology, University Hospital of Amiens, France

## Abstract

**Background:**

The best method to deliver intraperitoneal chemotherapy (IPC) for peritoneal carcinomatosis from ovarian cancer is not well defined. The aim of this study was to assess the ability of hyperthermia and adrenaline to enhance the intratumoral accumulation of cisplatin in a rat model of peritoneal carcinomatosis.

**Methods:**

Four groups of 5 BDIX rats with ovarian peritoneal carcinomatosis underwent IPC with 30 mg/l of cisplatin according to the following conditions: normothermia at 37° for 1 or 2 hours, hyperthermia at 42°C for 1 hour or normothermia at 37°C for 2 hours with 2 mg/l adrenaline. Tissue platinum content was measured by atomic absorption spectroscopy. The effect of hyperthermia, adrenaline and the duration of exposure to the drug was measured *in vivo *(tissue concentration of platinum in tumor, abdominal and extra abdominal tissues) and *in vitro *(cytotoxicity on human ovarian cancer cells).

**Results:**

*In vitro*, hyperthermia and longer exposure enhanced the accumulation and the cytotoxic effect of cisplatin on cancer cells. *In vivo*, only the 2 hours treatment with adrenaline resulted in increased platinum concentrations. The rats treated with adrenaline showed significantly lower concentrations of cisplatin in extra peritoneal tissues than those treated with hyperthermia.

**Conclusion:**

Adrenaline is more effective than hyperthermia in order to enhance the intratumoral concentration of cisplatin in rats with peritoneal carcinomatosis from ovarian origin. It may also decrease the systemic absorption of the drug.

## Introduction

Despite recent improvements, the prognosis of patients with peritoneal carcinomatosis from digestive or ovarian origin treated with systemic chemotherapy remains poor [1,2]. Intraperitoneal chemotherapy (IPC) improves the control of regional disease in ovarian cancer and increases survival in carcinomatosis of colorectal origin [3,4]. Trials have shown a survival benefit with post-operative IPC versus intravenous administration of cisplatin-based chemotherapy in ovarian cancer [5,6]. However, post-operative IPC showed poor tolerance and reduced quality of life in comparison with standard systemic chemotherapy [6]. Intraoperative IPC after cytoreductive surgery is a widely used alternative which achieves good results [7-9]. However, the best method for IPC has not yet been determined [10,11]. Heated intraperitoneal chemotherapy (HIPEC) with moderate hyperthermia (41°C to 43°C) is a potentially curative approach for peritoneal carcinomatosis [4]. Very encouraging results have been recently obtained with HIPEC using oxaliplatin at 43°C for 30 minutes in selected patients with carcinomatosis from colorectal origin [9]. As cisplatin is currently the most active systemic drug against ovarian carcinoma, it has also been used for HIPEC [12-16]. This technique is feasible, but somewhat toxic, and most people limit HIPEC with cisplatin to 1 hour at 42°C or 43°C. No randomized studies have compared heated with non-heated intraperitoneal cisplatin in ovarian carcinoma.

In previous papers, we reported that intraperitoneal adrenaline increased platinum uptake in rat peritoneal tumor nodules by a factor of 2 to 3 [17-19]. Adrenaline acts through vasoconstriction by limiting drug wash out from the peritoneal cavity. Animals treated with intraperitoneal cisplatin and adrenaline were definitively cured, whereas those treated with intraperitoneal cisplatin alone had only a delay in tumor growth [18]. In two phase I studies, intraperitoneal cisplatin with adrenaline was feasible in patients with refractory peritoneal carcinomatosis. We also established the maximal tolerated concentration of adrenaline (2 mg/l) in combination with 30 mg/l of cisplatin in two successive 1-hour peritoneal baths at 37°C after complete cytoreductive surgery [20,21]. However, the ability of hyperthermia and adrenaline to enhance the effect of cisplatin has never been compared. This was the aim of this experimental preclinical comparative study conducted in a rat model of peritoneal carcinomatosis.

## Methods

### Animals

Female inbred BDIX strain rats, 3 months old, weighing 200-250 g, were bred in constant conditions of temperature, hygrometry and exposure to artificial light. Experimental protocols followed the "Guidelines on the protection of experimental animals" published by the Council of the European Community (1986). The Burgundy's University Animal Care and Use Committee approved all of the procedures.

### Cancer cells and tumor model

A previously described rat model of peritoneal carcinomatosis was used. We previously reported the likeness of this rat model to human ovarian carcinomatosis in terms of peritoneal extension and chemo sensitivity to cisplatin [22]. The DHD/K12/TRb cell line originated from a dimethylhydrazine-induced colonic carcinoma in BDIX rats (ECACC N° 90062901). Its PROb clone was selected for its regular tumorigenicity when injected into syngenic rats [23]. PROb cells were maintained in Ham's F10 culture medium supplemented with 10% fetal bovine serum. SKOV-3 (HTB-77) and OVCAR-3 (HTB-161) human ovarian carcinoma cells originated from ATCC (Manassas, VA). IGROV-1 human ovarian carcinoma cells were a courtesy from Jean Benard, MD (Institut Gustave Roussy, Villejuif, France). The human ovarian cells were cultured in RPMI medium with 10% fetal bovine serum.

The cells were detached from the culture flask using trypsin and EDTA and centrifuged in the presence of complete culture medium with fetal bovine serum to inhibit trypsin. The PROb cells were suspended in 3 ml of serum-free Ham's F10 medium and then injected into the peritoneum of anesthetized rats (2 × 10^6 ^cells in each rat). The size of the peritoneal tumor nodules depended upon time.

### *In vitro *drug cytotoxicity assay

The PROb rat colon cancer cell line and the three human ovarian cancer cell lines (SKOV-3, OVCAR-3, and IGROV-1) were incubated *in vitro *with 30 mg/l cisplatin at 42°C for 1 hour, 37°C for 2 hours (in the presence or not of 2 mg/l adrenaline), or 37°C for 1 hour (control cells).

*In vitro *cytotoxicity of cisplatin on cancer cells was determined using a quantitative clonogenic assay. Cells (5 × 10^4^/well) were seeded and cultivated in 96-well tissue culture plates for 72 hours until confluence. Cell incubation with cisplatin was performed in serum-free Ham culture medium at 37°C or 42°C. After rinsing, the cells were trypsinized and seeded again in 24-well tissue culture plates. After 6 days of culture, the cells were washed with phosphate buffered saline, fixed with pure ethanol for 10 min, and then stained with 1% crystal violet in distilled water. After flushing the excess dye with water, the remaining dye was eluted with 33% acetic acid. The optical density (OD) was read on an automatic photometer at a wavelength of 540 nm. Cell survival was determined as the ratio of OD in treated wells to OD in control wells × 100. Experiments were done twice in triplicate.

### Treatment of animals

The rats were treated 21 days after intraperitoneal cell inoculation. Laparotomy was performed in anaesthetized rats (isoflurane inhalation as induction and then 100 mg/kg of intramuscular ketamine and 15 mg xylazine into the back leg for maintenance) to check the presence of a peritoneal carcinomatosis (present in 95% of animals). At day 21 after cell injection, the tumor nodules were confluent in the epiploic area and extended partly to the peritoneum wall, including nodules in the area of the diaphragm. The abdomen was then closed in such a way as to make it watertight. Twenty rats were distributed into 4 groups of treatment (5 rats per group), which are presented in Table 1.

**Table 1:** Characteristics of treatment in each group of rats.

Group	Cisplatin	Adrenaline	Temperature	Duration of treatment
**1**	30 mg/ml	No	37°C	1 h

(1 bis*)	30 mg/ml	2 mg/l	37°C	1 h

**2**	30 mg/ml	No	42°C	1 h

**3**	30 mg/ml	2 mg/ml	37°C	2 h (twice 1 hour)

**4**	30 mg/ml	No	37°C	2 h (twice 1 hour)

(*) In another experiment group 1 bis achieved the same tissue concentration of cisplatin as group 1 (unpublished data), thus this group was not repeated in the present study

The ***first group***(control group) received 30 mg/l of intraperitoneal cisplatin (Sigma-Aldrich, L'Isle d'Abeau, France) in 50 ml of saline solution (9 g/l NaCl) at 37°C. The ***second group***received HIPEC for 1 hour at 42°C with 30 mg/l of cisplatin. After laparotomy, an electronic thermal probe was placed in the epiploic area, an inward catheter above the right liver, and an outward catheter in the left splenic area. After watertight abdomen closure, a closed circuit was established by an electric pump (Abbott-Gemstar, Crestline Medical, Pleasant Grove, UT, USA) at a flow rate of 15 ml/min. Total volume of the circuit was 500 ml of saline solution which was pre-heated to 37°C. Starting time was defined as the moment the temperature reached 41.5°C and 30 mg/l cisplatin was added. The temperature was kept constant at 42°C for 1 hour in the peritoneal cavity by immersing an intermediate reservoir and about 1 meter of the circuit tubing in a thermostat-regulated bath at an average temperature of 48°C. The ***third group***had a 2 hours treatment with 30 mg/l of cisplatin and 2 mg/l of intraperitoneal adrenaline: after 1 hour the abdomen was open to empty the peritoneal cavity and a second identical bath was then performed for 1 additional hour. A previous experiment showed that 1 hour of treatment with 2 mg/ml adrenaline at 37°C did not increase the platinum content in peritoneal nodules and, thus, such a group was not planned in this study (unpublished data). The ***fourth group***underwent the same treatment as the third group, but without adrenaline. All animals from the 4 groups were kept anesthetized, lying on the back, for the entire duration of the treatment, using repeated IM ketamine and xylazine injections as necessary.

At the end of treatment, the rats were sacrificed; the abdominal cavity was opened and abundantly washed with water. Epiploic tumor nodules (200 mg), the left diaphragm, a piece of the muscle lining the abdominal cavity measuring 5 × 5 × 1 mm thick, parietal thoracic muscle (200 mg) in order to reflect the extra-abdominal tissues, half of the left kidney, and about 200 mg of the anterior edge of the liver were sampled and kept at -80°C until the platinum assay.

The comparison of groups 1 and 2 should assess the effect of hyperthermia; that of groups 3 and 4 should assess the effect of adrenaline; and that of groups 1 and 4 should assess the effect of the duration of IPC. A 2-hour HIPEC was impossible due to intolerance of the animals.

### Atomic absorption spectrometry

The total concentration of platinum was measured by atomic absorption spectrometry (AAS). Cultured cells were washed twice after cisplatin incubation, then trypsinised and counted. Cell pellets were frozen at - 80°C until AAS assay. After weighing, the frozen rat tissues were digested in a microwave digester (MLS-1200 Mega, Milestone, Sorisole, Italy). Platinum concentration was measured after dilution in distilled water, using a Zeeman atomic absorption spectrometer (Spectra-A; Varian, Les Ulis, France). Platinum is 65.01% of the molecular mass of cisplatin; to convert platinum concentrations into cisplatin concentrations, the first must be multiplied by 1.54.

### Statistical Analysis

Because of the small sample size, nonparametric tests were used to analyze the concentrations of platinum and the operative time. The Kruskal-Wallis test was performed to detect global statistically significant differences in the extent of platinum accumulation in the organs and tumors between the four groups. When a significant difference was found the Mann-Whitney test was used for 2 × 2 comparisons between groups. A two-tailed P value of\0.05 was considered significant for all tests. Data collection and statistical calculations were performed by SPSS (version 10.0) software (SPSS, Chicago, IL, USA).

## Results

### In vitro accumulation and cytotoxicity of cisplatin on cancer cells

**Figure 1:**
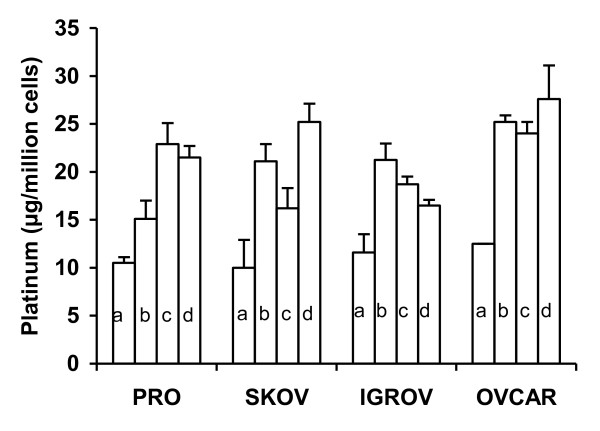
***In vitro *platinum accumulation in cancer cells**. Cells (1 × 10^6^/well) were seeded in 12-well culture plates for 72 hours then incubated with 30 mg/l cisplatin in serum-free Ham medium. Incubation conditions were: 1 hour at 37°C (a), 1 hour at 42°C (b), and 2 hours at 37°C without (c) or with (d) 2 mg/l adrenaline. Mean and SD of 3 determinations are represented.

A temperature of 42°C was toxic by itself. In comparison with the basal level, the number of residual adherent cells in the wells was reduced after 1 hour incubation at 42°C (decrease of percentage of 18%, 43%, 51%, and 17% for the PROb, SKOV-3, OVCAR-3, and IGROV-1, respectively). This was not the case after 2 hours of treatment with cisplatin with or without adrenaline at 37°C. Cellular platinum concentration was increased by hyperthermia in all cells (Figure 1). Extending the incubation to 2 hours also increased the platinum content in all cell lines, but there was no influence of adrenaline.

**Figure 2:**
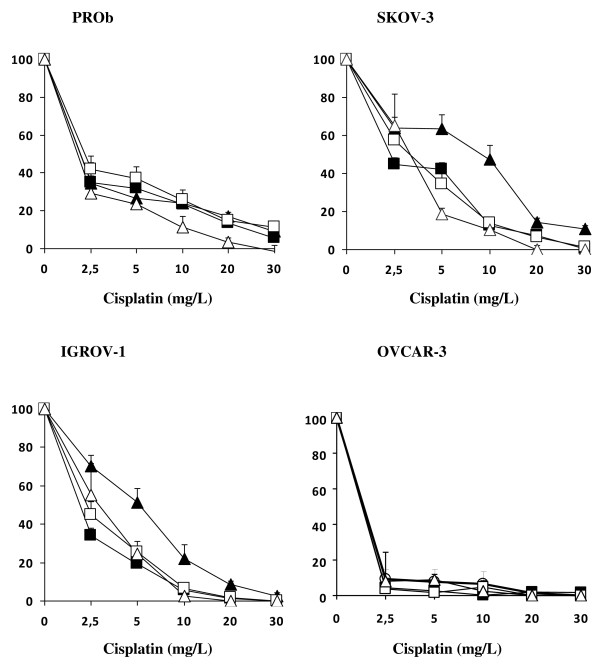
***In vitro *cytotoxicity of cisplatin**. Cells (5 × 10^4^/well) were seeded in 96-well culture plates for 72 hours, then treated with cisplatin in serum-free Ham medium. Treatment conditions were: 1 hour at 37°C (dark triangles), 1 hour at 42°C (open triangles), 2 hours at 37°C without (dark squares) or with (clear squares) 2 mg/l adrenaline. Mean and SD of 4 determinations of cell survival (percent of control cells) are represented.

Sensitivity to cisplatin depended on the cell lines (Figure 2). The most sensitive line was OVCAR-3 (IC 50 less than 2.5 mg/l after 1 hour incubation at 37°C), whereas the least sensitive lines were SKOV-3 and IGROV-1 (IC 50 ranging between 5 and 10 mg/l). The rat PROb cell line had intermediate sensitivity to cisplatin (IC 50 2.5 mg/l). A concentration of 30 mg/l cisplatin was found to be almost complete cytotoxic (≥90%) for all cell lines. This concentration was chosen for the *in vivo *experiments. The cell toxicity of cisplatin was significantly enhanced by 1 hour of hyperthermia at 42°C for the resistant SKOV-3 and IGROV-1 cell lines, but not for the sensitive OVCAR-3 and PROb cells. Cisplatin cytotoxicity was also enhanced by extending the incubation time to 2 hours; the improvement in cytotoxicity was of the same order as that achieved by 1 hour of hyperthermia.

### Platinum accumulation in rat peritoneal nodules and organs

In the hyperthermia group, the closed circuit made it possible to reach a stable intra-abdominal temperature (42.1°C ± 0.46°C) in a mean time of 15.5 minutes (range 4-21 minutes) with variations of less than 0.5°C along the procedure. Temperature was dependent on the flow rate and was unstable at a flow of less than 15 ml/min.

Tolerance to HIPEC was poor. Only 3 out of 5 rats survived until the end of the experiment. The others presented an abnormal respiratory rhythm at about 45 minutes and died before the end. This precluded the performance of a 2-hour HIPEC. In contrast, all of the animals that were treated at 37°C, for either 1 or 2 hours, with or without adrenaline, were alive and well at the end of the experiment.

**Figure 3:**
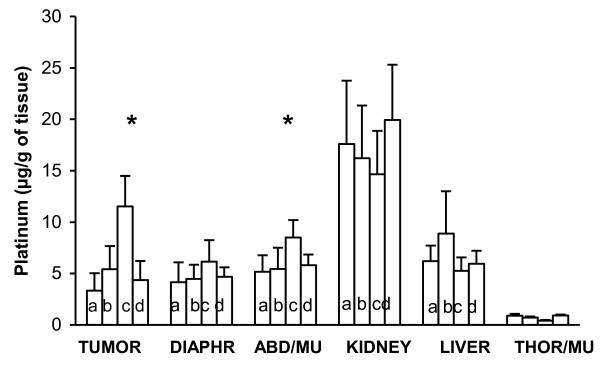
***In vivo *accumulation of platinum in peritoneal tumor and organs**. Intraperitoneal chemotherapy was performed using 30 mg/l of cisplatin. Tumor and organs were sampled: after 1 hour cisplatin at 37°C (a), after 1 hour cisplatin at 42°C (b), after 2 hours cisplatin with (c) or without (d) 2 mg/l adrenaline. Mean and SD of 5 animals. Asterisk indicates a statistical difference (p < 0.01) between the 2 hours treatment at 37°C with 2 mg/L adrenaline, and the 1 hour treatment at 42°C. ABD/MU = abdominal muscle and THOR/MU = thoracic muscle.

Platinum concentrations in rat organs and peritoneal nodules were measured according to the different treatments (Figure 3). Regarding the platinum content in peritoneal nodules, the difference between group 1 (control, 1 hour IPC), and groups 4 (2 hours IPC) or 2 (HIPEC) did not reach significance (p = 0.06 and 0.19, respectively). In contrast, a 3-fold increase in tumor platinum content was found in group 3 (adrenaline) as compared to groups 1 (control, p = 0.005) and 2 (HIPEC, p = 0.005). Platinum concentration in the abdominal muscle lining the peritoneal cavity was also significantly greater in group 3 (adrenaline) as compared to group 4 (HIPEC) (p = 0.006), but did not reach significance in the diaphragm (p = 0.08).

Out of the peritoneal cavity (kidney and thoracic muscle), the accumulation of platinum was lower in group 3 (adrenaline) than in groups 1 (control) and 4 (HIPEC) (p = 0.05 and p = 0.001, for the kidney and the thoracic muscle, respectively).

## Discussion

The present study reports the greater uptake of platinum in peritoneal nodules and in peritoneum lining muscle when adrenaline was used in combination with cisplatin, as compared to HIPEC. This underlines the interest of adrenaline to increase the tissue concentration of chemotherapy and the fact that the best method to deliver of IPC remains to be defined [10,17,21].

The rats treated with adrenaline (group 3) received this treatment for 2 hours, as compared to those undergoing HIPEC (group 2) during only 1 hour. A 1-hour adrenaline group was not performed because a previous unpublished experiment found no significant difference after this treatment as compared to the control group. A 2-hour HIPEC was impossible due to intolerance of the animals to such a procedure. It could be argued that the longer exposure explains the higher tissue uptake of cisplatin. However, group 4 had a 2 hours IPC and did not achieved significantly better concentrations than group 1 (1 hour IPC); the difference was close to significance (p = 0.06), but it can not explain a 3-fold increase in concentration. The effect of time probably exists, but is small. This is consistent with the results of a previous pharmacokinetic study which showed that most of the uptake happens at the beginning of IPC, when the gradient of concentrations is higher: a twice 1-hour bath (as done in the present study) with a newly prepared identical solution was more effective than a 2-hour bath [24]. Similar results have been obtained in HIPEC with oxaliplatin [11].

Adrenaline also increased the drug content in the muscle of the abdominal wall. We observed a ratio of 5 to 17 in drug uptake between an abdominal muscle and a distant thoracic muscle. This reflects the pharmacological advantage of IPC to obtain high local drug concentrations in the abdominal wall, peritoneum and muscle lining, all of which are possibly infiltrated by malignant cells in peritoneal carcinomatosis. In previous studies we used a higher concentration of adrenaline (5 or 10 mg/L) [18,19]. In the present study it was reduced according to a recent phase I clinical trial, which established the safety of 2 mg/l of adrenaline, whereas 3 mg/l induced cardiovascular collateral effects (tachycardia, arterial hypertension or electric signs of cardiac ischemia) [21].

Despite their longer exposure, rats treated with adrenaline showed lower extraperitoneal concentrations of platinum than both, the control and the HIPEC groups. This is probably explained by the vasoconstrictor effect of adrenaline which prevented the systemic diffusion, and thus, the potential toxicity of cisplatin. At the opposite, HIPEC has been shown to increase systemic absorption of chemotherapy drugs due to heat-induced vasodilatation [11].

Our results confirmed the well-known enhancing effect of hyperthermia on the platinum uptake, as well *in vitro *as *in vivo *[25-28]. *In vitro*, the thermal enhanced ratio (TER) after 1 hour exposure at 42°C compared to 37°C ranged from 1.5 to 2.1, depending on the cell line. The TER was lower than that found in other studies (3.4 for 1 hour at 43°C in a different colon cancer cell line in rats; 2.2 or 3.9 for hamster kidney cells and Chinese hamster fibroblasts, respectively) [26,27]. The reasons for these discrepancies (technical variations or true differences in membrane permeability in different cell lines) are unknown. The increased accumulation due to extending exposure to 2 hours (1.6 to 2.5) was of the same order as the TER recorded after 1 hour. Temperature is mainly thought to accelerate the passive diffusion of cisplatin by disturbing the phospholipid bilayer arrangement, even if other mechanisms, such as a direct apoptotic or necrotic effect, may be involved in cell death.

*In vitro *experiments on cancer cell lines alone cannot predict the *in vivo *effect of temperature or adrenaline. Tumor tissue penetration is the limiting factor for the activity of the chemotherapeutic agents [29]. It has been hypothesized that the depth of penetration of cisplatin could be increased by hyperthermia through its effects on convection and diffusion in tissues, increasing cell uptake of the drug, tumor blood flow and vascular permeability. Despite the clinical development of HIPEC with platinum compounds, only a few studies have been done in order to establish the basis of this technique. Two contradictory studies have been reported in rat models of peritoneal carcinomatosis [27,30,31]. Differences in the hyperthermia technique could explain this discrepancy. Los et al. immersed the whole animal in a thermostatically controlled water bath, resulting in whole-body hyperthermia rather than locoregional hyperthermia [27]. This could have modified both blood concentrations and vascular permeability, and may explain why plasmatic cisplatin was about 3 times greater at 41°5 than at 38°C and why platinum content was about twice as great in all organs, including the extra-abdominal organs such as the lung. Our technique allowed us to heat only the abdominal cavity. Using this method of heating, a 1-hour HIPEC at 42°C did not increase platinum content in the peritoneal tumor nodules or in the peritoneal wall lining. Abdominal hyperthermia was poorly tolerated by the animals; sometimes it was even necessary to stop the procedure before 60 minutes. This poor tolerance made it impossible to compare the two methods in terms of survival. Our negative results on HIPEC with cisplatin are consistent with those obtained by other authors using similar methods [31,32]. An explanation of this negative result could be the temperature-related increase in blood flow through the peritoneal nodules and the peritoneum due to local vasodilatation and resulting in an increase in the wash out of the cisplatin [33].

In contrast with heat, adrenaline at a concentration of 2 mg/l for 2 hour achieved a 2 to 3-fold increase in platinum content in the peritoneal tumor nodules. Such an increase boosts the cytotoxic effect of cisplatin *in vitro *(Figure 2). Previous rat experiments have shown us that 2 hours of IPC are required to observe the enhancing effect of adrenaline [17,19], and our following clinical trials have taken into account this parameter [20,21].

Experimental data show that adrenaline is more effective and better tolerated than hyperthermia in order to enhance the penetration of cisplatin. It also minimizes the systemic absorption of cisplatin. Hyperthermia was not well tolerated in this rat model, but it is in humans. Future clinical trials performing IPC with cisplatin for ovarian carcinoma should compare the effectiveness of adrenaline and hyperthermia in order to improve the effect of intraperitoneal chemotherapy.

The authors declare that they have no competing interests.

## Authors' contributions

OF, FR and DD carried out the *in vivo *experiments. SL and HT carried out the *in *vitro experiments. BC participated in the design of the study and performed the statistical analysis. POD, FG and PR conceived the study, and participated in its design and coordination. All authors read and approved the final manuscript.
